# A putative new *Besnoitia* species in the southern black-eared opossum *Didelphis aurita*

**DOI:** 10.1016/j.ijppaw.2024.100998

**Published:** 2024-09-21

**Authors:** Juan Pablo Arrabal, Gastón Moré, María Marcela Orozco, Elisa Helman, Juliana Notarnicola, Walter Basso, Bárbara Betina Hartmann, Andrea Schapira, Leonardo Minatel

**Affiliations:** a Instituto de Biología Subtropical IBS-CONICET, Universidad Nacional de Misiones- UNaM, Av. Tres Fronteras 183, Puerto Iguazú, CP 3370, Misiones, Argentina; b Consejo Nacional de Investigaciones Científicas y Técnicas (CONICET), Ciudad Autónoma de Buenos Aires, Buenos Aires, Argentina; c Laboratorio de Inmunoparasitología (LAINPA), Facultad de Ciencias Veterinarias, Universidad Nacional de La Plata, Calle 60 y 118, (B1904), La Plata, Buenos Aires, Argentina; d Institute of Parasitology, Vetsuisse Faculty, University of Bern, Länggassstrasse 122, Bern, 3012, Switzerland; e Instituto de Ecología, Genética y Evolución de Buenos Aires (IEGEBA-CONICET), Facultad de Ciencias Exactas y Naturales, Universidad de Buenos Aires, Av. Int. Guiraldes, (C1428EGA), Ciudad Autónoma de Buenos Aires, Argentina; f Grupo de Bioestadística Aplicada (GBA), Instituto de Cálculo Numérico Rebeca Cherep de Guber, Facultad de Ciencias Exactas y Naturales, Universidad de Buenos Aires (FCEyN-UBA), Intendente Güiraldes 2160, Ciudad Universitaria, Edificio 0 + Infinito (C1428EGA), Buenos Aires, Argentina; g Universidad de Buenos Aires, Facultad de Ciencias Veterinarias, Chorroarín 280, (C1427CWO), Ciudad Autónoma de Buenos Aires, Argentina

**Keywords:** *Besnoitia* sp., *Didelphis aurita*, Argentina, Infection, PCR, Histopathology

## Abstract

*Besnoitia* spp. are cyst-forming coccidian parasites with a broad host range, infecting various wild and domestic animal species. Northamerican opossums (*Didelphis virginiana*) are severely affected by the infection with *B. darlingi*. This study presents a case of infection with *Besnoitia* in a road-killed female southern black-eared opossum (*Didelphis aurita)* in Misiones, Argentina. Many 0.5–1 mm cysts were observed in several muscles and visceral organs and were microscopically identified in skeletal muscles, tongue, and heart. Histological analysis disclosed multiple spherical cysts with a myriad of bradyzoites like-cells and a well-defined cyst wall. A small number of degenerate and ruptured cysts, surrounded by mild to moderate inflammation were observed. Genomic DNA from an individual cyst and muscle was extracted and ITS1 marker and *18S rRNA* gene fragments from sarcocystid protozoa were successfully amplified by PCR and sequenced. The *18S* sequence exhibited 100% identity with sequences of *B. darlingi* and *B. oryctofelisi*. Comparison of the complete ITS1 sequence (259 bp) revealed an identity of 99.2% with *B. oryctofelisi* and 97.7% with *B. darlingi.* This result together with the phylogeny positioning, suggest that the *Besnoitia* sp. in the present case differ from *B. darlingi*, being closely related with *B. oryctofelisi*.

## Highlights

• Numerous 0.5–1 mm *Besnoitia* tissue cysts found in a *Didelphis aurita* from Argentina.
• The obtained ITS1 sequence differed from *B. darlingi* from Northamerican opossums.
• *Besnoitia* sp. recorded in this work could be a new species.
• *Besnoitia* spp. from the Americas seems to have a common ancestor.

## Graphical abstract

**Figure:**
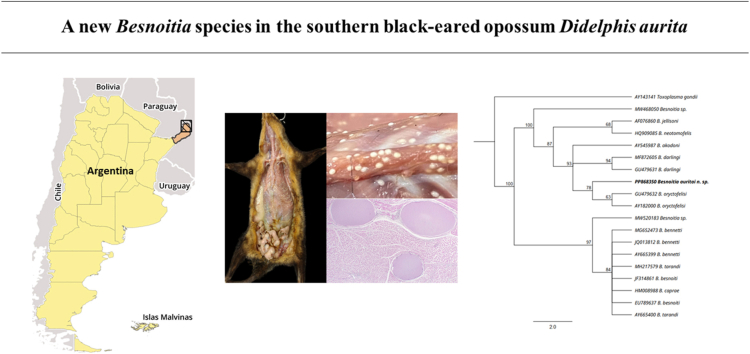

## 1 Introduction

The southern black-eared opossum, *Didelphis aurita*, inhabits the Atlantic Forest in South America. It is distributed in southeastern Brazil, from Bahia to Rio Grande do Sul, eastern Paraguay and northeastern Argentina, in northern and central Misiones province (Gardner, 2007). This species is frequent in continuous and remnant native forests associated with streams (Massoia et al., 2012; Chemisquy et al., 2019). It is less frequent in anthropic modified environments than its sympatric close related White-eared opposum (*Didelphis albiventris)* (Cruz et al., 2019). Roadkill poses a substantial threat to different species within protected natural areas of the Atlantic Forest, both in Brazil and Argentina. The southern black-eared opossum is among the most frequently affected mammals by roadkill in northern Misiones (Chemisquy et al., 2019). Despite its association with remnant native forests and frequent roadkill incidents, there is no evidence suggesting that southern black-eared opossum is at risk of conservation concern (Chemisquy et al., 2019; Cruz et al., 2019). It is currently listed as "Least Concern" in the International Union for Conservation of Nature's Red List of Threatened Species (Astúa et al., 2021).

Related to the parasites and pathogens occurring in southern black-eared opossum, numerous potentially pathogenic agents have been identified in this host in Brazil, such as *Mycoplasma* sp. (Oliveira et al., 2023), *Salmonella enterica* (Casagrande et al., 2011), *Ehrlichia* sp. (Guimarães et al., 2008), among others. Moreover, several protozoans and helminths have been detected in the southern black-eared opossum, including *Sarcocystis* spp., *Toxoplasma gondii*, *Schistosoma mansoni*, and *Cruzia tentaculata* (Casagrande et al., 2009; Costa Neto et al., 2019; Bezerra-Santos et al., 2021). In Argentina, Hartmann (Hartmann, 2023) mentioned the presence of seven helminths, including the nematodes *Capillaria* sp., *C. tentaculata*, Cyclophyllidea, the trematode *Rhopalias* sp. and two acanthocephalans. These findings underline the importance of understanding the role of these marsupials in wild cycles of pathogens and assessing their role as potential reservoirs and transmitters of parasites in the region. *Besnoitia* is a genus of cosmopolitan apicomplexan protozoans included in the family Sarcocystidae. Different *Besnoitia* species parasitize a broad intermediate host range, such as cattle, equids, rodents, opossums, among others, developing tissue cysts (Dubey and Yabsley, 2010). Felids are definitive hosts for some *Besnoitia* species, in which the parasites undergo a sexual multiplication in the intestine, leading to the production and shedding of oocysts (Dubey et al., 2003a; Dubey and Yabsley, 2010; Smith and Frenkel, 1977, 1984; Verma et al., 2017; Wallace and Frenkel, 1975). Currently, at least 10 species of *Besnoitia* are described; however, only in a few of them, the complete life cycles are known (Basso 2018). In North and South America, different intermediate hosts have been documented, such as the virginian opossum (*Didelphis virginiana)* for *B. darlingi*, domestic rabbits (*Oryctolagus cuniculus*) for *B. oryctofelisi,* and different rodent species for *B. akodoni*, *B. jellisoni* and *B. neotomofelis* (Dubey et al., 2003a, 2003b; Dubey and Yabsley, 2010; Venturini et al., 2002). Domestic cats (*Felis catus*) can serve as definitive hosts for *B. neotomofelis*, *B. darlingi*, *B. oryctofelisi* and *B. wallacei* (Olias et al., 2011). In the case of *B. darlingi*, bobcats (*Lynx rufus*) were shown to be natural definitive hosts (Verma et al., 2017). *Besnoitia* oocysts are morphologically similar to *T. gondii* oocysts and can be easily misidentified (Dubey and Yabsley, 2010). Given that the different *Besnoitia* species cannot be differentiated based on cyst morphology, molecular methods are necessary for its identification (Venturini et al., 2002; Basso, 2018; Schares et al., 2020). The *ITS1* region is highly conserved within each *Besnoitia* species and is being used as the main target for molecular identification techniques (Schares et al., 2020).

In Argentina, *Besnoitia*-like cysts have been previously documented in various hosts, including those from *B. oryctofelisi* in domestic rabbits (Venturini et al., 2002), and from undescribed *Besnoitia* species in pichis (*Zaedyus pichiy*) (Superina et al., 2009) and in vizcachas (*Lagostomus maximus*) (Cwirenbaum et al., 2021).

In wild mammals, besnoitiosis typically manifests with cysts in the skin, skeletal muscle, and visceral organs, accompanied by acute and chronic inflammatory processes (Jack et al., 1989; Elsheikha et al., 2003; Juan-Salles et al., 2004; Shaw et al., 2009; Superina et al., 2009). Several studies suggest that factors such as young age, immunological naivety, immunosuppression, and stress may predispose individuals to clinical disease (Glover et al., 1990; Ellis et al., 2012). This study documents a putative new species of *Besnoitia* in southern black-eared opossum, incorporating histopathological, and molecular aspects.

## 2 Materials and methods

### 2.1 Study area

**Fig. 1:**
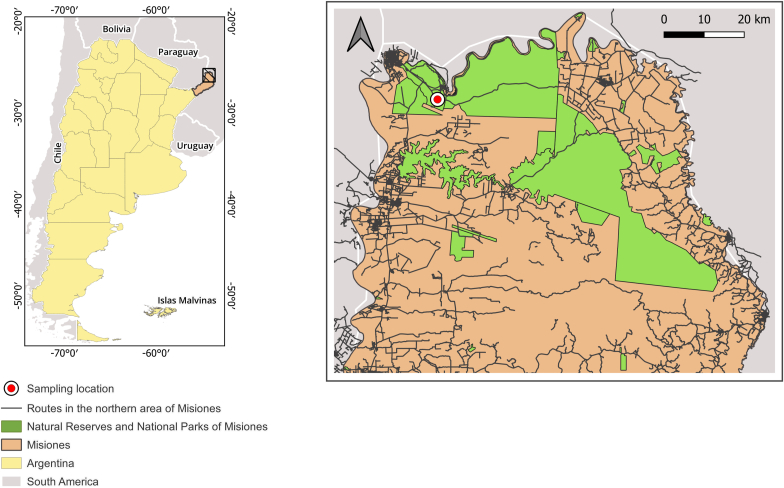
Map of Argentina showing Misiones province (left). Detailed area of Northwest Misiones (right); red dot shows the point where the road-killed southern black-eared opossum (*Didelphis aurita)* was found in the National Route 101 traversing the National Park Iguazú (green). (For interpretation of the references to color in this figure legend, the reader is referred to the Web version of this article.)

The sampling was carried out in the Iguazú National Park (PNI), located in the department of Iguazú, northwest of the province of Misiones, Argentina (Fig. 1). The area is bordered by Brazil to the north and Paraguay to the west. In addition, the area is known to receive more than one million tourists from all over the world who visit the PNI every year. The PNI is included in the Upper Paraná Atlantic Forest ecoregion and is part of the largest continuous remnant of Atlantic Forest in the world (25° 55′ 52.32″ S; 54° 15 '30.60″ W). Despite its highly fragmented state, the Misiones rainforest remains one of the most biologically diverse ecosystems in the world (Cullen et al., 2001) and hosts the most varied mammalian community in Argentina (De Angelo et al., 2008). Within this diverse community, several carnivorous species may prey on didelphids, including the cougar (*Puma concolor*), jaguar (*Panthera onca*), ocelot (*Leopardus pardalis*), jaguarundi (*Herpailurus yagouaroundi*), margay (*Leopardus wiedii*), southern tiger cat (*Leopardus guttulus*), crab-eating fox (*Cerdocyon thous*), and greyheaded tayra (*Eira barbara*) (Presley, 2000; Wang, 2002; Facure et al., 2003; Moreno et al., 2006; Tófoli et al., 2009; Perilli et al., 2016).

The vegetation is a subtropical semi-deciduous forest composed of different plant communities, such as gallery forests, bamboo forests, forests dominated by palmettos (*Euterpe edulis*), araucaria forests (*Araucaria angustifolia*), among others (Di Bitetti et al., 2003; Galindo-Leal and de Gusmão Câmara, 2003). This area is characterized by an altitude of 220 m and has a subtropical climate, with annual rainfall ranging between 1700 and 2100 mm and an average annual temperature of 20 °C (Ligier, 2000).

### 2.2 Animal samples

A female wild southern black-eared opossum was retrieved deceased following a vehicular collision on National Route 101 (Misiones, Argentina), a roadway traversing the PNI, on August 25th, 2023 (Fig. 1). The carcass was handled and sampled in accordance with protocols sanctioned by the technical office of the National Parks Administration (IF-2023-34961534-APN-DRNEA#APNAC), adhering to the guidelines proposed by the WOAH (World Organisation for Animal Health) for wildlife disease surveillance (https://www.woah.org/app/uploads/2021/09/a-wildlifehealth-conceptnote.pdf). A comprehensive dataset detailing parameters such as sex, reproductive status, age category, weight, and body condition score was recorded. Subsequently, a complete necropsy was conducted, scrutinizing each body cavity for anomalies, and systematically documenting the macroscopic characteristics of each organ.

Tissue samples for histopathology, including skeletal muscle, heart, tongue, lung, liver, spleen, kidney, intestines, diaphragm, lymph nodes, fetuses, brain, and cerebellum, were collected and preserved in 10% formalin. Preservation included both a segment of healthy tissue and, when present, a segment of any identified lesions. A pooled sample comprising muscular tissues, was stored at −80 °C and subsequently prepared for DNA extraction.

### 2.3 Histopathological studies

Formalin-fixed tissue samples were processed by standard histologic techniques using a Leica TP1020 tissue processor. Briefly: tissues were embedded in paraffin, sections of 4-μm-thick were prepared using a Leica RM2245 microtome, which were stained with hematoxylin and eosin. Slides were examined by optical microscopy and lesions were characterized. Photographs were obtained with a Leica DM750 microscope and a Leica ICC50W camera.

### 2.4 Molecular identification and phylogenetic analysis

DNA was extracted using a commercially available kit, according to the manufacturer's instructions (ADN PuriPrep-T kit; Inbio-Highway, Argentina) from pooled skeletal muscles containing several cysts and one individual cyst retrieved from skeletal muscle. For *18S rRNA* gene, a fragment of around 650 bp was amplified by conventional PCR, using SarcoF and SarcoR primers (Moré et al., 2011). Another conventional PCR, targeting the ITS1 and flanking regions was performed using primers SU1F and 5.8SR2 (Gjerde, 2014). Both PCRs were carried out as previously described by Bentancourt Rossoli et al. (2023). Each PCR routine included a negative control (DNA extraction process control sample), a no template control (NTC, ultrapure water), and a positive control (*S. miescheriana* DNA for *18S rRNA* and *S. rileyi* DNA for ITS1 PCR). A GeneAmp PCR System 9700 cycler (Applied Biosystems) was used to perform all PCR assays. Amplification products were examined after electrophoresis in a 1.5% agarose gel stained with ethidium bromide and photographed with a UV light image system (E-Box, Vilber, France). Obtained amplicons were excised from the gels and purified using a commercial kit according to the manufacturer's instructions (Zymoclean™ Gel DNA recovery Kit, Zymo Research, USA) and submitted for Sanger sequencing to Microsynth, Balgach, Switzerland (https://srvweb.microsynth.ch), along with both primers used for each PCR. Sequences obtained were analyzed and aligned using the Geneious Prime software (https://www.geneious.com). The consensus sequences with trimmed primers were compared with the GenBank database by BLAST analysis (http://blast.ncbi.nlm.nih.gov/Blast.cgi). The obtained ITS1 sequence was aligned with sequences of *Besnoitia* spp. and a distance tree was constructed using Bayesian inference with gamma rate variation and HKY85 substitution model (MrBayes plugin, Geneious Prime software). A ITS1 sequence from *Toxoplasma gondii* (AY143141) was used as an outgroup. All the sequences used for the phylogenetic tree are listed in Table 1. The obtained *18S rRNA* fragment sequence was also aligned with other *Besnoitia* spp. sequences (which have at least 90% coverage with our sequence) and a phylogenetic tree was constructed using the same model and software mentioned before. A *18S rRNA* sequence from *Toxoplasma gondii* (OR805035) was used as an outgroup to root the tree.

**Table 1:** Besnoitia spp. ITS1 sequences from different hosts used for constructing the phylogenetic tree. The *Toxoplasma gondii* sequence was used as outgroup. ∗obtained from cell culture derived parasites.

Coccidian species	Host	GenBank Accession	Locality	Author
*Toxoplasma gondii*	not indicated	AY143141	USA	Su et al. (2003)
*Besnoitia* sp.	*Acinonyx jubatus*	MW468050	Namibia	Schares et al. (2021)
*B. jellisoni*	not indicated∗	AF076860	Australia	Ellis et al. (2000)
*B. neotomofelis*	*Neotoma micropus*	HQ909085	Texas, USA	Charles et al., 2012
*B. akodoni*	*Akodon montensis*	AY545987	Brazil	Dubey et al. 2003a,b
*B. darlingi*	*Lynx rufus*	MF872605	Mississippi, USA	Verma et al. (2017)
*B. darlingi*	*Didelphis virginiana*	GU479631	Mississippi, USA	Dubey et al. 2003a,b
*Besnoitia* sp.	*Didelphis aurita*	PP868350	Misiones, Argentina	This study
*B. oryctofelisi*	*Oryctolagus cuniculus*	GU479632	Argentina	Rosenthal et al. (2016)
*B. oryctofelisi*	*Oryctolagus cuniculus*	AY182000	Argentina	Dubey et al. 2003a,b
*Besnoitia* sp.	*Equus asinus*	MW520183	Italy	Villa et al. (2021)
*B. bennetti*	*Equus asinus*	MG652473	Belgium	Liénard et al. (2018)
*B. bennetti*	*Equus asinus*	JQ013812	Pennsylvania, USA	Ness et al. (2012)
*B. bennetti*	*Equus asinus*	AY665399	Michigan, USA	Elsheikha et al., 2005
*B. tarandi*	*Rangifer tarandus caribou*	MH217579	Quebec, Canada	Schares et al. (2019)
*B. besnoiti*	*Bos taurus*	JF314861	Bologna, Italy	Gentile et al. (2012)
*B. caprae*	*Capra aegagrus hircus*	HM008988	Iran	Namazi et al. (2011)
*B. besnoiti*	*Bos taurus*	EU789637	Spain	Fernández-García et al., 2009
*B. tarandi*	*Rangifer tarandus*	AY665400	Finland	Dubey et al. (2004)

## 3 Results

### 3.1 Macroscopic examination

**Fig. 2:**
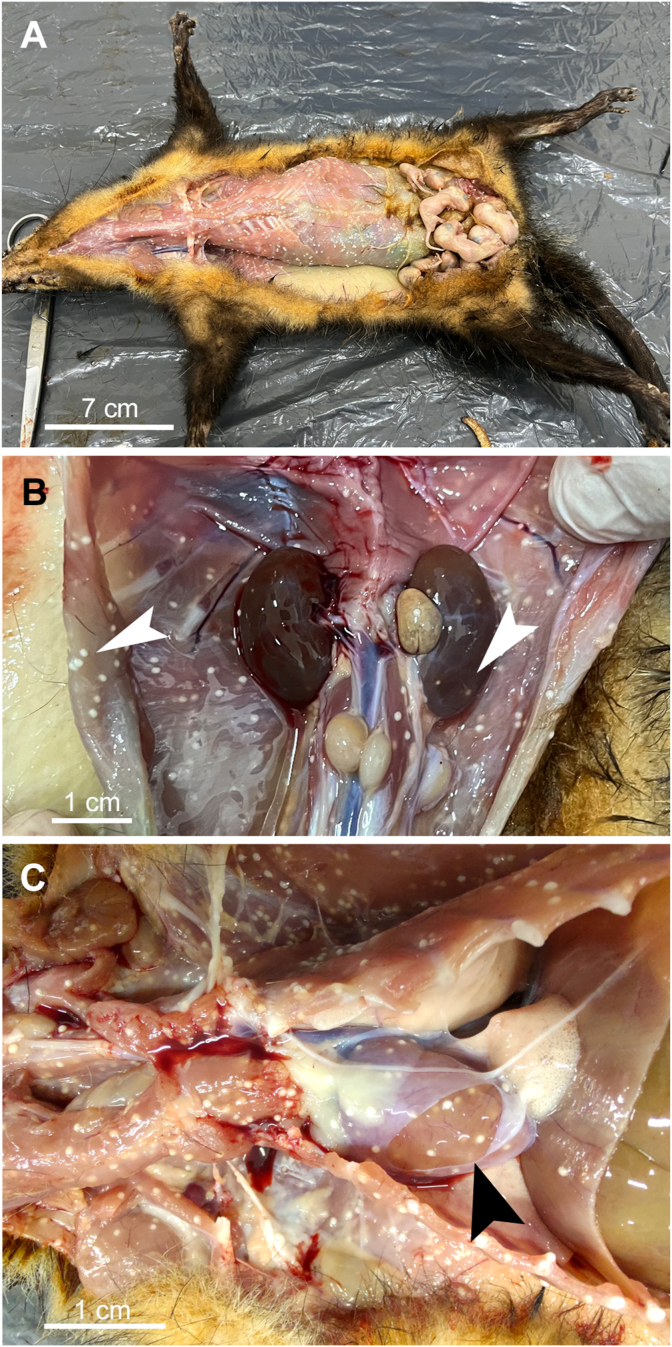
Dissection of a female southern black-eared opossum (*Didelphis aurita)*. Multiple whitish cysts in skeletal muscles (**A**), in diaphragm and kidney (white arrowheads) (**B**), and heart (black arrowhead) (**C**).

The specimen collected was an adult female of southern black-eared opossum, weighing 790 g, with eight embryos in the marsupium (Fig. 2a). Macroscopic examination revealed the presence of a huge number of macroscopic whitish cysts in various organs of the adult female, including subcutaneous tissue, teats, fasciae, skeletal muscles, heart, liver, kidneys, and spleen; however, skeletal muscles and heart were the most affected organs (Fig. 2). The cysts measured between 0.5 and 1 mm in diameter and were predominantly located superficially, although some were detected deep within the tissues, particularly in skeletal muscle and heart (Fig. 2b and c). Based on fat reserves, muscle mass, visible bony prominences, and organ shape, the body condition of the animal was considered good.

### 3.2 Histopathological findings

**Fig. 3:**
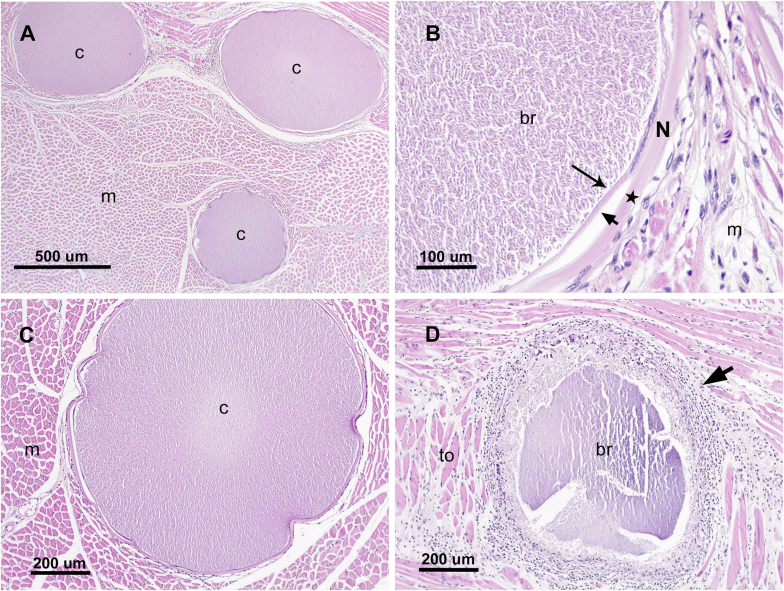
Histopathological sections of *Besnoitia* sp. cysts in muscles stained with hematoxylin and eosin. Cysts with bradyzoites (c) in skeletal muscles (m) (**A**). Detail of a cyst wall in the heart muscle (m), composed of three layers: capsule (star), host cell cytoplasm (short arrow), and parasitophorous vacuole (long arrow) with a myriad of bradyzoites (br). Note the compressed nucleus of the host cell (N) (**B**). Higher magnification of a *Besnoitia* sp. cyst (c) in skeletal muscle (m) showing absence of tissue reaction (**C**). Degenerated *Besnoitia* sp. cyst on the tongue (to) surrounded by severe inflammation (short arrow) (**D**).

Histopathological analysis revealed multiple cysts, located mostly in skeletal muscle, tongue, and heart of the adult female. No cysts were found in the fetuses. The cysts were spherical, measured up to 1000 μm, and had a 10–20 μm thick-three-layered wall. The outermost layer was an eosinophilic and hyalinized capsule of collagen fibers. The next visible layer was the cytoplasm of the host cell; sometimes compressed host cell nuclei could be seen in it. The inner layer was a thin parasitophorous vacuole, which contained a myriad of approximately 2 × 5 μm basophilic bradyzoites-like cells (Fig. 3a and b). Most of the cysts had no tissue reaction (Fig. 3c). Some cysts were surrounded by a mild number of lymphocytes, macrophages, plasma cells, and eosinophils. There were a small number of degenerated and ruptured cysts, surrounded by mild to moderate inflammation, composed by macrophages, lymphoid cells, eosinophils, and giant cells (Fig. 3d). Other findings were multifocal bronchointerstitial pneumonia, with intralesional nematodes; diffuse pleocelular esophagitis with intralesional nematodes; and reactive lymphoid tissue in spleen and lymph nodes. No other lesions were found in the rest of the organs examined. Three slides with histopathological sections from muscle, tongue and heart containing *Besnoitia* sp. cysts were stored at the Helminthological Collection Museo de La Plata, La Plata, Buenos Aires, Argentina (code MLP-Pr 105).

### 3.3 Molecular characterization and phylogenetic positioning of a putative new *Besnoitia* sp.

The two samples processed were positive by the *18S rRNA* PCR and the amplicons were suitable for subsequent sequencing. The obtained sequences (625bp, primers trimmed) from muscles and one individual cyst, were identical between them, and exhibited 100% identity and coverage with previously described sequences of *B. darlingi* (MF872605) and *B. oryctofelisi* (GU479632).

By ITS1 PCR the sample from the individual cyst showed a more concentrated product and was sequenced, resulting in a consensus sequence (with trimmed primers) of 484bp (including complete ITS1, and *18S rRNA* and *5.8S* flanking regions). The identity was 98.56% (100% coverage) with *Besnoitia darlingi* (MF872605) and 99.54% (89% coverage) with *Besnoitia oryctofelisi* (GU479632). When only the complete ITS1 sequence (259bp) was considered, the percentage of identity was 99.23% (two single nucleotide polymorphisms-SNPs) with *B. oryctofelisi* sequences (GU479632 and AY182000) and 97.7% (4 SNPs and 2 gaps) with *B. darlingi* sequences (GU479631, HQ163919, AF489696), all with 100% coverage.

**Fig. 4:**
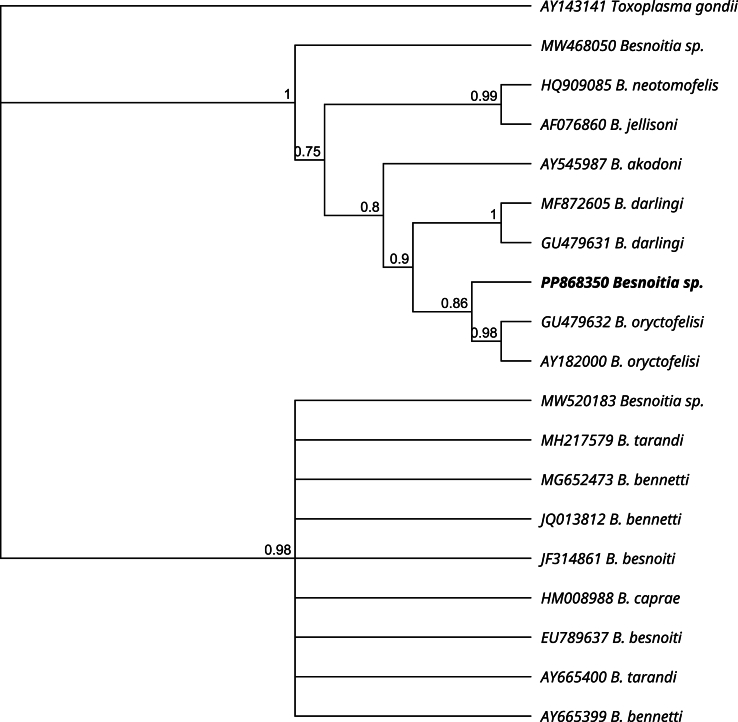
Phylogenetic distance tree using Bayesian inference with gamma rate variation and HKY85 substitution model (MrBayes plugin, Geneious Prime software) of ITS1 sequences from *Besnoitia* spp. A ITS1 sequence from *Toxoplasma gondii* (AY143141) was used as an outgroup. Branches are labelled with the posterior probability. The sequence obtained in the present study appears in bold.

**Fig. 5:**
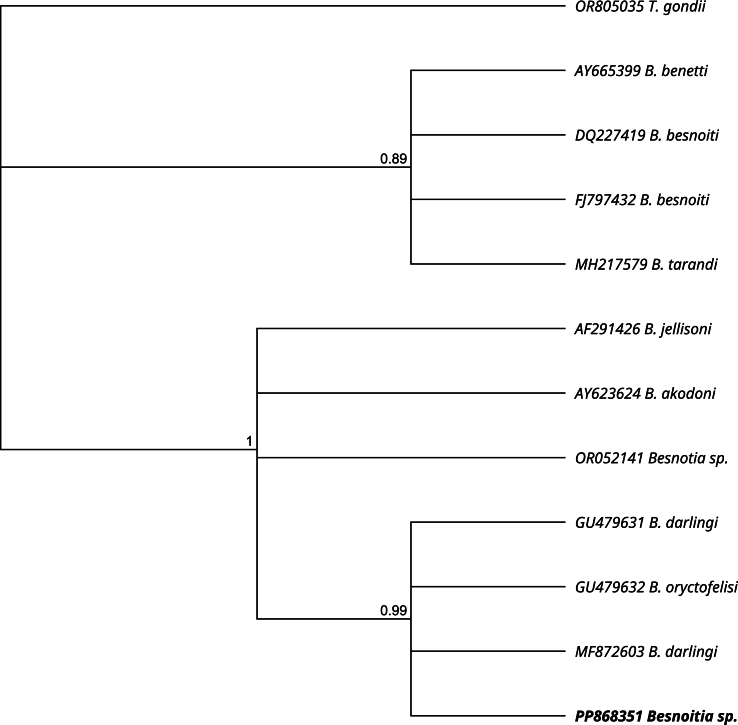
Phylogenetic distance tree using Bayesian inference with gamma rate variation and HKY85 substitution model of *18S rRNA* fragment sequences from *Besnoitia* spp. A *18S rRNA* sequence from *Toxoplasma gondii* (OR805035) was used as an outgroup. Branches are labelled with the posterior probability. The sequence obtained in the present study appears in bold.

The obtained sequences of complete ITS1 containing *18S* and *5.8S rRNA* gene flanking regions (Accession number PP868350) and *18S rRNA* gene fragment (Accession number PP868351) were registered in the GenBank. In the phylogenetic tree (Fig. 4) our sequence is positioned on a branch closely related to B. *oryctofelisi*, and as close relatives in a sister group appear *B. darlingi* and *B. akodoni* sequences. The phylogeny using *18S rRNA* sequences positioned the sequence of *Besnoitia* sp. from southern black-eared opossum together with sequences of *B. darlingi* and *B. oryctofelisi* and closely related to *B. akodoni*, *B. jellisoni* and a *Besnoitia* sp. from rodents (Fig. 5).

## 4 Discussion

This study documented a putative new *Besnoitia* species from southern black-eared opossum in Misiones, Argentina, which was characterized by molecular analyses. In Argentina, *Besnoitia* parasites had so far only been recorded in three mammal species: domestic rabbits (Venturini et al., 2002), Patagonian pichis (Superina et al., 2009) and wild vizcachas (Cwirenbaum et al., 2021). In this sense, southern black-eared opossum is the third native wild mammal documented as a host of *Besnoitia* in Argentina.

In the present study, *Besnoitia* sp. showed preference for muscular and cardiac tissues in the hosts, similar to previous reports from other *Besnoitia* spp. (Juan-Salles et al., 2004; Superina et al., 2009; Ellis et al., 2012). Virginia opossum severely infected with *B. darlingi* showed a general deteriorating condition with multiple round, firm, white cysts in the ear pinnae, lips, tongue, retina and iridal tissue, skeletal muscle, as well as in the myocardium, liver, kidneys, lungs, spleen and other organs (Ellis et al., 2012; Gardner, 2019). In this study, the most largely affected tissues were skeletal muscle, heart and tongue and the animal presented a good body condition. The observation of a high number of large cysts suggests an advanced stage of chronic infection. Histopathological analysis confirmed the presence of cysts exhibiting the typical characteristics of *Besnoitia* (Venturini et al., 2002; Shaw et al., 2009). The majority of the cysts showed minimal tissue reaction, suggesting a modulation of the host immune response, a phenomenon commonly observed in chronic infections by some sarcocystid parasites (Frenkel, 1989). This is also consistent with observations done in rabbits and vizcachas infected with *Besnoitia* sp. in Argentina, where no severe cellular damage or inflammation was recorded in the tissues surrounding the cysts (Venturini et al., 2002; Cwirenbaum et al., 2021). The inflammation surrounding degenerated and ruptured cysts may indicate acute episodes of localized immune response, possibly induced by the release of parasitic antigens (Superina et al., 2009). In pichis and maras, the infection with *Besnoitia* sp. has been associated with acute and chronic pulmonary inflammations (Juan-Salles et al., 2004; Superina et al., 2009); however, the broncho-interstitial pneumonia observed in our case appears to be related to a nematode infection, as no protozoan cysts were found in the lungs. Similarly, pulmonary oedema and congestion with intralesional nematodes have been previously reported in Virginia opossum co-infected with *B. darlingi* (Ellis et al., 2012).

So far, besnoitiosis in opossums was assumed to be caused by *B. darlingi*. However, accurate species identification in intermediate hosts can only be reliably achieved through molecular methods (Schares et al., 2020). In our study, the obtained *18S rRNA* sequences showed 100% identity with both *B. darlingi* and *B. oryctofelisi* sequences. However, when using ITS1, a target with a higher discriminative power (Schares et al., 2020), it was revealed that our sequence had a higher identity with *B. oryctofelisi* (but with 2 SNPs) and differed extensively from *B. darlingi* (4 SNPs and 2 gaps). Moreover, in the cladogram performed with Bayesian inference using ITS1 sequences, *Besnoitia* sp. is placed as a sister group from *B. oryctofelisi* found in rabbits from Argentina, and both *Besnoitia* species are closely associated with *B. akodoni* from the sigmodontine rodent *Akodon montensis* from Brazil, and from *B. darlingi* isolated from Virginia opossum and a bobcat in USA. In addition, *B. neotomofelis* from the Southern Plains woodrat (*Neotoma micropus*), *B. jellisoni* from the white-footed deer mouse (*Peromyscus maniculatus*) from the USA, and *Besnoitia* sp. oocysts shed by a cheetah in Namibia are phylogenetically closer related to this group as to other *Besnoitia* species affecting ruminants and equids (Fig. 4). These genetic similarities and phylogenetic placement suggest that all *Besnoitia* species detected in rodents, marsupials, rabbits as intermediate hosts or felids as definitive hosts could have a common ancestor, as previously suggested by Oyarzún-Ruiz et al. (2023). Interestingly, almost all these species have been described in the Americas.

Besnoitiosis in opossums has been increasingly linked to severe debilitation and mortality, with factors such as youth, immunosuppression, and stress possibly linked to enhanced susceptibility to development of clinical manifestations (Ellis et al., 2012). The extensive presence of cysts within vital organs or ocular tissues can predispose them to predation and compromised foraging abilities (Gardner, 2019), as might have happened to the specimen under scrutiny in this study. However, the specimen was in apparent good body condition, with no external injuries.

The study of road-killed specimens offers valuable access to internal anatomical insights, shedding light on dietary habits, parasitic fauna, and pathologies, being an important resource for opportunistic surveillance. Previous investigations in the same region as the present study, have provided essential insights into the zoonotic and ecological significance of parasites, revealing novel parasite-host cycles in Argentina (Arrabal et al., 2017, 2020, 2023; Maldonado et al., 2019). In this study, a putative new species of *Besnoitia* was identified, which prompts further investigation into the natural transmission cycle in the area. Evidence suggests that the domestic cat may not serve as the optimal definitive host for certain *Besnoitia* species, with low intensity of oocyst excretion noted in *B. neotomofelis, B. darlingi,* and *B. oryctofelisi* (Olias et al., 2011). Notably, bobcats have been identified as natural definitive hosts for *B. darlingi*, underlining the potential role of wild felids in the life cycle of *Besnoitia* parasites (Verma et al., 2017; Schares et al., 2020). The Atlantic Forest harbors six species of wild felids, with opossums serving as significant dietary complements, depending on the felid species (Crawshaw Jr, 1995; Tófoli et al., 2009; Bianchi et al., 2011; dos Santos et al., 2022). It is possible to assume that some of these felids could be definitive hosts for the *Besnoitia* sp. Identified here. Nevertheless, further studies are needed to identify the hosts and the identity of this potentially new *Besnoitia* species.

## Funding

The molecular studies were made possible by internal funding from the Institute of Parasitology of the 10.13039/100009068University of Bern, Switzerland.

## CRediT authorship contribution statement

**Juan Pablo Arrabal:** Writing – review & editing, Writing – original draft, Project administration, Methodology, Investigation, Conceptualization. **Gastón Moré:** Writing – review & editing, Writing – original draft, Resources, Methodology, Formal analysis. **María Marcela Orozco:** Writing – review & editing, Writing – original draft, Supervision, Project administration, Methodology, Investigation, Conceptualization. **Elisa Helman:** Writing – original draft, Visualization, Methodology. **Juliana Notarnicola:** Writing – review & editing, Visualization. **Walter Basso:** Writing – review & editing, Resources, Formal analysis. **Bárbara Betina Hartmann:** Writing – original draft. **Andrea Schapira:** Methodology. **Leonardo Minatel:** Writing – review & editing, Visualization, Methodology, Investigation, Conceptualization.

## Declaration of competing interest

We, Arrabal Juan Pablo, Moré Gastón, Orozco María Marcela, Helman Elisa, Notarnicola Juliana, Basso, Walter, Hartmann Bárbara Betina, Schapira Andrea, and Minatel Leonardo, authors from the manuscript intitled “A new *Besnoitia* species in the southern black-eared opossum *Didelphis aurita*” report no conflict of interest.
